# Recalculation of dose for each fraction of treatment on TomoTherapy

**DOI:** 10.1259/bjr.20150770

**Published:** 2016-02-05

**Authors:** Simon J Thomas, Marina Romanchikova, Karl Harrison, Michael A Parker, Amy M Bates, Jessica E Scaife, Michael PF Sutcliffe, Neil G Burnet

**Affiliations:** ^1^ Cambridge University Hospitals NHS Foundation Trust, Department of Medical Physics and Clinical Engineering, Addenbrooke's Hospital, Cambridge, UK; ^2^ Cancer Research UK VoxTox Research Group, University of Cambridge, Addenbrooke's Hospital, Cambridge, UK; ^3^ Cavendish Laboratory, Department of Physics, University of Cambridge, Cambridge, UK; ^4^ Department of Oncology, Cambridge University Hospitals NHS Foundation Trust, Addenbrooke's Hospital, Cambridge, UK; ^5^ Department of Oncology, University of Cambridge, Cambridge Biomedical Campus, Addenbrooke's Hospital, Cambridge, UK; ^6^ Department of Engineering, University of Cambridge, Cambridge, UK

## Abstract

**Objective:**

The VoxTox study, linking delivered dose to toxicity requires recalculation of typically 20–37 fractions per patient, for nearly 2000 patients. This requires a non-interactive interface permitting batch calculation with multiple computers.

**Methods:**

Data are extracted from the TomoTherapy^®^ archive and processed using the computational task-management system GANGA. Doses are calculated for each fraction of radiotherapy using the daily megavoltage (MV) CT images. The calculated dose cube is saved as a digital imaging and communications in medicine RTDOSE object, which can then be read by utilities that calculate dose–volume histograms or dose surface maps. The rectum is delineated on daily MV images using an implementation of the Chan–Vese algorithm.

**Results:**

On a cluster of up to 117 central processing units, dose cubes for all fractions of 151 patients took 12 days to calculate. Outlining the rectum on all slices and fractions on 151 patients took 7 h. We also present results of the Hounsfield unit (HU) calibration of TomoTherapy MV images, measured over an 8-year period, showing that the HU calibration has become less variable over time, with no large changes observed after 2011.

**Conclusion:**

We have developed a system for automatic dose recalculation of TomoTherapy dose distributions. This does not tie up the clinically needed planning system but can be run on a cluster of independent machines, enabling recalculation of delivered dose without user intervention.

**Advances in knowledge:**

The use of a task management system for automation of dose calculation and outlining enables work to be scaled up to the level required for large studies.

## INTRODUCTION

Patients having curative treatment with radiotherapy receive radiation dose over a number of separate attendances known as “fractions” of radiotherapy. The radiotherapy treatment is planned using a kilovoltage (kV) CT image set, onto which a clinician outlines target volumes and organs at risk. The internal anatomy of the patient varies from day to day, therefore the dose delivered each day will vary from the dose determined at planning. Where a patient is being treated with daily image-guided radiotherapy on a TomoTherapy^®^ system (Accuray Inc., Madison, WI), a megavoltage (MV) image is taken before treatment to determine the position of the patient; this is matched against the planning kV CT images, and the patient's position is adjusted by means of couch movements and gantry rotations (shifts in *x*/*y*/*z* and roll) to minimize geometric uncertainties.

For prostate radiotherapy, a number of authors have found variation in daily rectal position and differences between planned and delivered doses to the rectum.^1–5^ These studies support the idea that accumulated dose over the course of treatment may differ from the planned dose.^6^ When determining the relationship between effects such as normal tissue toxicity and dose, it is essential to have a means of estimating the dose actually delivered.

This work forms part of the VoxTox Programme,^7^ which is funded by Cancer Research UK and is part of the National Institute for Health Research Clinical Research Network portfolio. The ultimate aim of VoxTox is to establish the accumulated dose and its relationship with toxicity, in approximately 2000 participants treated for prostate cancer, head and neck cancer or a central nervous system tumour. All patients have been treated using helical tomotherapy using daily MV image guidance.^8^ The study was granted ethics approval by the Essex Research Ethics Committee in February 2013. All participants had given informed consent.

The TomoTherapy Planned Adaptive^®^ module^9^ allows dose to be recalculated based on the MV CT. However, this application has a graphical user interface and ties up the use of a planning terminal that is in daily use for routine clinical work. For the purpose of this study, we require recalculation of dose for typically 20–37 fractions per patient, for approximately 2000 patients, which in total therefore requires in excess of 60,000 fractions to be recalculated. For our preliminary study on ten patients,^5^ it was possible to perform this by manually reoutlining and using an interactive dose calculation system. Scaling up to hundreds of patients requires a non-interactive interface that permits batch calculation with multiple computers. We have developed a system for automatic recalculation of daily delivered dose, which can be integrated within a batch calculation framework derived from the one used in particle physics.^10^ We describe results of calculation times applying this system to the first 151 patients with prostate cancer in this study.

## METHODS AND MATERIALS

Patients were treated on two TomoTherapy HiArt^®^ machines. The dose calculation algorithm used is based on that described by Thomas et al.^11^ Data are extracted from the TomoTherapy archive using methods developed in-house. The files extracted include the digital imaging and communications in medicine (DICOM) RTPLANs, the kV CT images, the MV images acquired before treatment on each fraction and the shifts (*x*, *y*, *z* and roll) applied for each fraction. All data are converted into the DICOM RT format. The shifts that are applied for each fraction of radiotherapy are determined on the basis of the matching, performed by the radiographers, between the MV CT images and the planning kV CT images to ensure that the correct target is covered by the radiation distribution. The system produces an initial automatic registration, which is manually adjusted by the radiographers to ensure soft-tissue matching. For recalculation of delivered dose, it is necessary to use the final moves applied, by applying the shift values from the co-ordinates of the in-plane isocentre values and applying the superior–inferior (SI) shift value to the couch position of the MV images. The roll is corrected for in-treatment by offsetting the gantry angle of each projection by the roll. This has the effect of rotating the dose distribution relative to the MV image.

The length of the MV scan was chosen to cover the length of the prostate, to enable daily image-guidance whilst minimizing imaging dose and time. As a result, the MV image set was shorter than the planning kV set. Imaging was usually carried out using the “coarse” settings which gives slices at 6-mm spacing.

**Figure 1.**
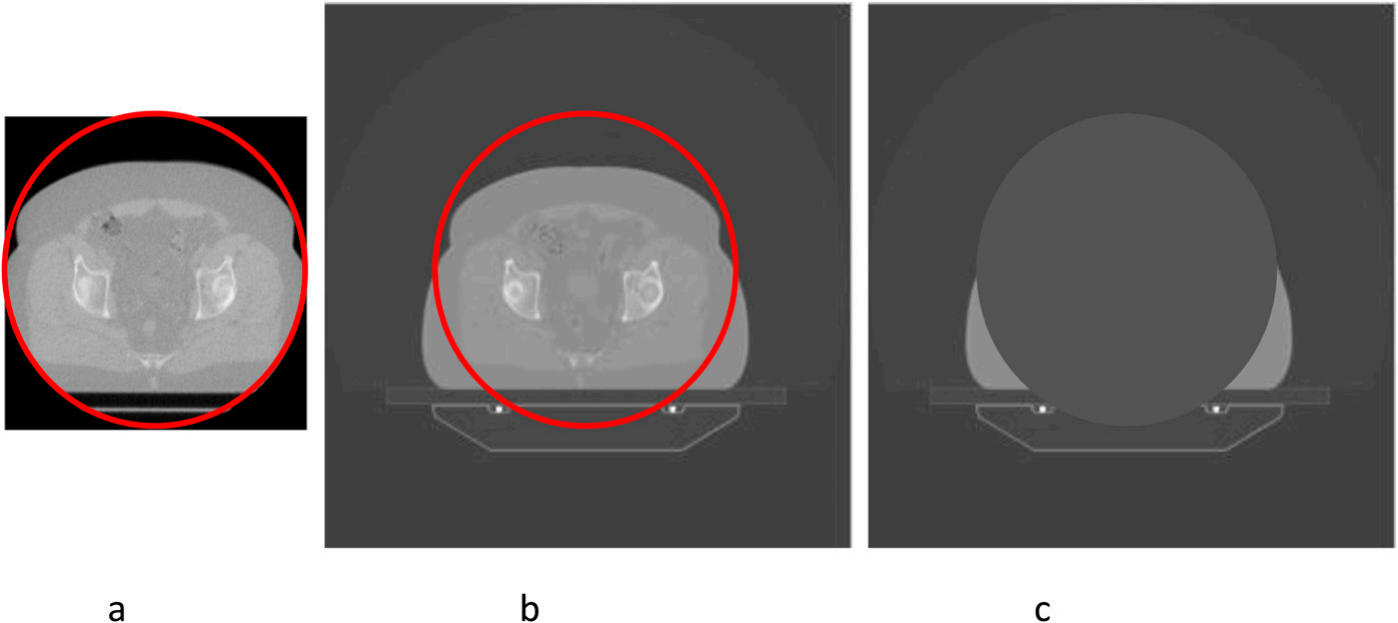
Combination of kilovoltage (kV) CT and megavoltage (MV) CT images. (a) The MV CT image, with the scan circle shown in red. (b) This circle superimposed on the aligned kV CT image. (c) The masked image, in which the area within the scan circle has been assigned zero density. The radiological path length to each point is the sum of the radiological path lengths from the first and third images. For colour see online.

The scan circle of the MV CT is 38.6 cm in diameter, which is frequently too small to cover the edge of the patient, as shown in Figure 1. It is therefore necessary to utilize kV CT in areas outside the MV CT scan circle, masked and transformed in a manner that allows for the shifts and roll that are applied.

All Hounsfield unit (HU) values within this circle are set to −1000 (corresponding to zero density) in the kV image. Figure 1 shows the process required to combine the two images. Where image matching required a roll to be applied, we offset the gantry angle of all control points in the dose calculation by this roll, exactly mimicking the situation in treatment delivery. Since the purpose of image matching is to ensure that dose is not rotated relative to the planning kV CT image, it is necessary to rotate the kV CT to compensate for this roll.

The ray-tracing algorithm described by Thomas et al^11^ is carried out twice, once for each image. The radiological path length to each point is the sum of the radiological path lengths from the MV CT and from the masked kV CT images. The two images have very different pixel sizes, and require different HU to electron density conversions. Producing a composite image, by interpolation and replacement of HU values, would require more computational resources (calculation time and storage space) than is required to ray trace twice through separate images. For MV images, the HU to electron density conversion varies over time. The calibration of MV CT images on TomoTherapy has not been stable over time.^12–14^ Prior to 2011, the TomoTherapy HiArt used a rotating target. The target required frequent replacement, which caused step changes in HU calibration with each target change, in addition to random fluctuations. TG-148^15^ recommends that monthly HU calibration tests should verify that water-equivalent materials vary by <30 HU from the calibrated image value to density table to maintain a dosimetric uncertainty of 2% or less. Since 2011, two changes introduced by Accuray Inc. have improved this: the use of a non-rotating target with a much longer lifetime and the introduction of a weekly user-run MV CT recalibration procedure. Measurements were performed using the density inserts (Gammex RMI Middleton, WI) inside the cylindrical phantom from TomoTherapy Inc. commonly referred to as the “cheese phantom”.^15^ We have repeatedly measured the phantom with density inserts over an 8-year period, at intervals varying between monthly and quarterly, and recorded the mean HU within each of a set of regions corresponding to the density inserts. The inserts had electron density relative to water of 0.30, 0.49, 0.942, 0.979, 1.018, 1.053, 1.080, 1.104, 1.112, 1.274, 1.472 and 1.694. Two additional inserts contained air and distilled water.

### Implementation

The files extracted from the archive were tokenized to remove patient identifiers before exporting from the hospital to the university department of physics. The files were stored in a hierarchical structure to facilitate their processing using the computational task-management system GANGA,^10^ which was originally developed for use on the ATLAS and LHCb experiments at CERN.^16^ GANGA is an extensible system for defining and managing arbitrary computing tasks (called “jobs”). Applications consist of a set of algorithms run in sequence; an application may run the same algorithm multiple times, with different configurations. A job definition consists of the algorithm to be run, the data set specifying patient data for processing, the backend specifying where to perform processing, plus handling options for splitting jobs into subjobs and for merging outputs of subjobs.

The files extracted include the treatment plan data, the kV CT scan (downsampled by TomoTherapy from 512 × 512 to 256 × 256, and with the CT couch replaced by the TomoTherapy couch), the MV images acquired before treatment on each fraction, the positional corrections (*x*, *y* and *z* shifts and roll) applied for each fraction and the planned dose data. All data were saved in the DICOM RT format following the TomoTherapy DICOM conformance statement.^17^ The shift and roll information were added to the MV image headers using private DICOM tags. Where an MV image does not immediately precede a treatment (*e.g.* in the event of a machine breakdown or where excessive rectal filling necessitated the patient going to the lavatory and being rescanned), a tag is added to the header indicating that this image should not be used for recalculation. Also stored are the RTSTRUCT objects (DICOM RT structure sets) of outlines drawn in the planning process, plus any RTSTRUCT objects associated with each MV image set, produced either by manual outlining or automatic segmentation.^5^

The automatic segmentation tool was written in MATLAB^®^ (MathWorks^®^, Natick, MA), using MATLAB'S two-dimensional implementation of the Chan–Vese algorithm.^18^ The HU values were rescaled to highlight the critical regions, with regions of air identified separately and rescaled to match the HU of rectal material. The starting contour for each slice was based on the equivalent contour in the planning scan. At the inferior end of the rectum, the contrast between the rectum and muscles is not sufficient to distinguish these consistently, even if viewed by an experienced oncologist. This lack of contrast leads the algorithm to overcontour on these slices, by including muscles near the rectum. This is detected by identifying the most superior slice with an unexpectedly large contour and replacing contours from the inferior end to this slice with the planning scan contours. In a small proportion of cases, incorrect outlining occurs on single slices; to reduce the incidence of this, contours were automatically compared with a smoothed three-dimensional surface formed from all contours in a scan; where a large difference was found, the contours were replaced by the corresponding smoothed contour. The use of the smoothing would typically improve conformity indices (measured against a test set of manually drawn contours) from 0.7 to 0.8. Key parameters in the autocontouring algorithm were tuned using a training set of data, using manual scanning as the gold standard for comparison and a testing set of data used to verify the accuracy of the autocontouring. Further details of the algorithm, including results of agreement with manually outlined contours, are given by Sutcliffe et al.^19^

**Figure 2.**
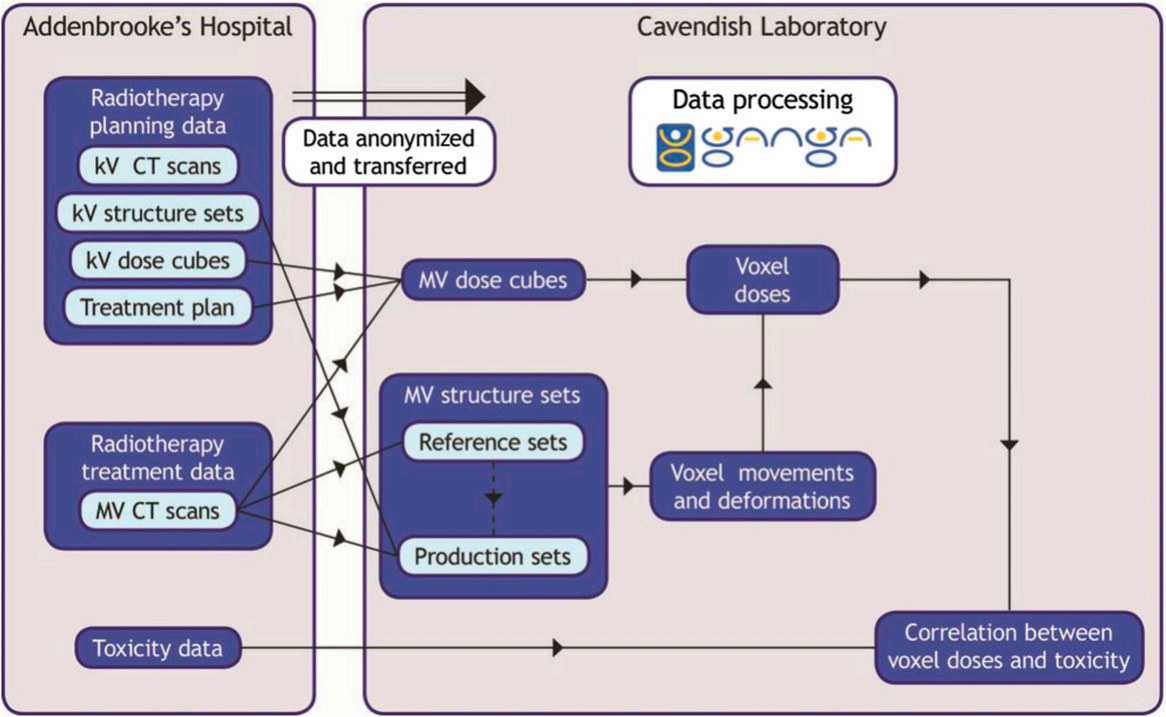
VoxTox data flows. kV, kilovoltage; MV, megavoltage.

Figure 2 gives an overview of the data flows in VoxTox. The dose calculation program was written as a command-line application using MATLAB. Interactive user input was replaced by automatically produced text files to deploy the program using GANGA. The calculation is performed over a set of points covering each MV CT image. To reduce calculation times, an option to downsample the grid spacing by a factor of 2 or 4 (or higher powers of 2 for testing) is provided. The pixel size of an MV CT image is typically 0.76 mm; fields of view for planning CT vary between 55 and 70 cm, hence the pixel size of a kV image is typically 1.05–1.37 mm, which after downsampling in TomoTherapy's dose calculation gives dose calculation grid spacings of 2.1–2.73 mm. Positioning dose calculation points at twice the MV CT spacing thus gives a finer resolution than the TomoTherapy dose, whereas positioning them at four times the spacing gives slightly coarser resolution. The downsampling applies only in-plane; the SI dose grid spacing is determined by the interval between MV images, which is 6 mm. The beam model used in dose calculation is as described by Thomas et al,^11^ using seven subprojections per projection.^20^

The calculations were performed on the Cambridge High-Energy Physics cluster, which has the ability to provide up to 240 job slots. The dose calculations were performed on eight dedicated batch machines. Four of these had Intel^®^ Xeon X560 processors (Intel, Santa Clara, WI) at 2.67 GHz, with each machine having 24 cores, 32 GB of memory and 12 job slots. The other four machines had Intel Xeon E5-2650 v. 2 processors at 2.60 GHz, with each machine having 16 cores, 64 GB of memory and 16 job slots.

The calculated dose cube is saved as a DICOM RTDOSE object. This RTDOSE object is then available to be read by utilities that perform dose accumulation or calculate other objects such as dose surface maps (DSMs).^21^ To demonstrate this functionality, we have implemented code for the calculation of DSMs based on the algorithms described by Murray et al^22^ and Buettner et al,^23^ by considering the rectum as a cylinder and sampling the dose at a set of equally spaced points on each MV CT slice. The cylinder was “cut” at the point where a vertical line from the centroid of each outline crossed the posterior edge and unfolded. The DSMs were summed over all the fractions, based on the SI positions of each image corrected for the shifts applied at treatment. Since the method of normalization removes variations of circumference from day to day, and assuming that the region of the rectum nearest to the prostate is relatively immobile (*i.e.* no overall rotations), summation of DSMs preserves spatial information in a way that summation of dose–volume histograms does not. Since the length of the MV CT image set was less than that of the rectum, the structures and doses from the planning CT were used at the top and bottom of the rectum. The DSMs were used to calculate generalized equivalent uniform doses (EUDs) using the formula of Niemierko.^24^

## RESULTS AND DISCUSSION

### Megavoltage image calibration

**Figure 3.**
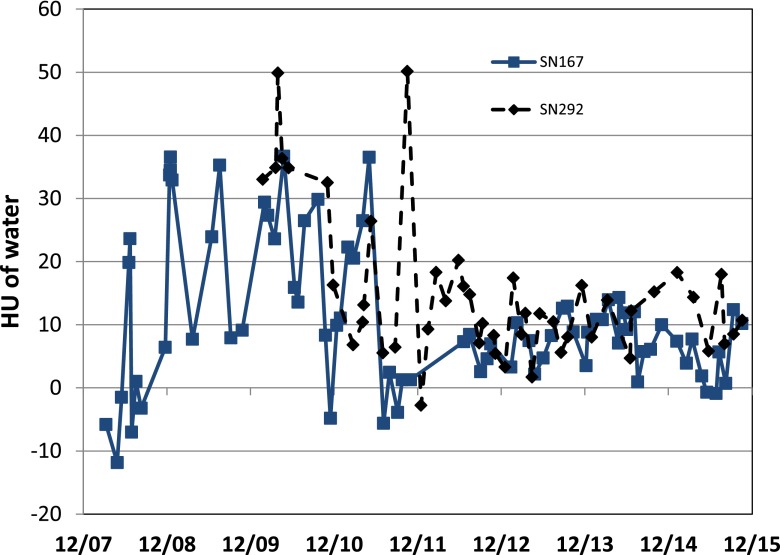
Hounsfield unit (HU) of water for Hi-Art SN167 from 2008 to 2014. The abrupt improvement in stability follows two changes by TomoTherapy: the replacement of the rotating target linear accelerator (linac) by a fixed target linac, and the introduction of a new weekly megavoltage CT calibration procedure.

Figure 3 shows the variation of HUs of a water insert from the dates of machine installation (end of 2007 and end of 2009) to 2015.

It can be seen that up to 2011, there was considerable variation between measurements, although still within the ±30 HU recommended by TG148.^15^ Following the introduction of the fixed target in 2011 and the weekly HU calibration procedure in 2012, this variation considerably decreased, such that it is now <±10 HU. Hence for results from 2008 to 2011, variations in HU calibration could be introducing uncertainties in dose of up to 2% whilst since 2011 this has reduced to <1%. If recent calibration data were to be used to recalculate doses from early in the life of the machine, they would erroneously show a systematic underdosing, since they would be systematically overestimating densities.

The change in weekly HU calibration procedure introduced in July 2012, as well as making the results more stable, caused a systematic reduction in the slope of the line of electron density *vs* HU as shown in Table 1. It should also be noted that there is considerable variation in slope between two machines. This is in contrast to the results observed for diagnostic kV CT scanners,^25^ where for low-*Z* materials, an offset of zero and a slope of 1000 appeared to fit a wide range of CT scanners.

**Table 1.** Offsets and slopes of Hounsfield unit (HU) calibration

Machine installed 2007			Machine installed 2009		
From	Offset	Slope	From	Offset	Slope
31/10/2007	25 ± 15	1000 ± 55	14/01/2010	35 ± 9	982 ± 27
29/07/2011	12 ± 15	992 ± 41	01/04/2011	15 ± 14	962 ± 33
01/07/2012	8 ± 4	970 ± 12	01/07/2012	10 ± 5	940 ± 10

Uncertainties of ±1 standard deviation are shown on the offsets and slopes, based on the variation of the mean HU value of water and 1.694 inserts over the relevant time period. The offset and slope used in the dose recalculation is chosen from this table on the basis of the date of the image. The values given have been used for all dates from the date given in the “from” column to the date given in the row below. The values in the final row are still valid in November 2015.

### Dose calculation

Table 2 shows the central processing unit time to calculate a dose cube for a single fraction of radiotherapy, memory requirements and size of output file for downsampling factors from 4 to 1. A downsampling factor of 2 has been chosen for subsequent calculations.

**Table 2.** Mean central processing unit (CPU) time, memory requirements and output size for calculating a dose cube, for downsampling factors from 4 to 1

Downsampling factor	CPU time (s)	Memory (MB)	Output size (kB)
4	1892 ± 262	449 ± 11	424 ± 32
2	8388 ± 1406	659 ± 28	1684 ± 128
1	33,001 ± 4208	1377 ± 71	6724 ± 511

In each case, results are from processing data for a single fraction for each of the 33 patients. Processing was performed on machines that have 24-core Intel^®^ Xeon X5650 CPUs (Intel, Santa Clara, WI), with a clock speed of 2.67 GHz. A downsampling factor of 2 gives an in-plane spatial resolution of approximately 1.5 mm.

**Figure 4.**
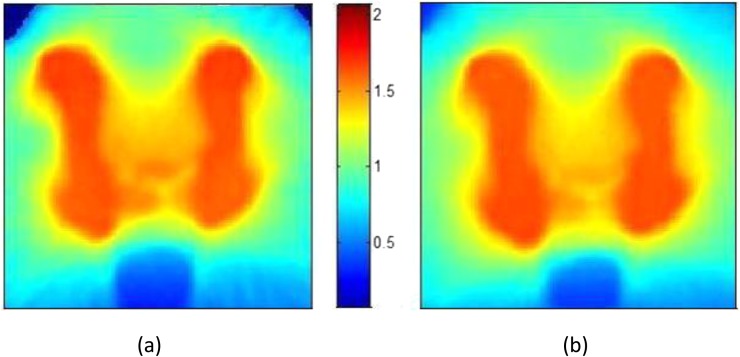
Comparison of dose calculations. (a) The dose calculated by the algorithms described in this article; (b) the dose calculated by TomoTherapy's planned adaptive module. The colour bar shows doses in Gray. The differences in the top corners result from differences in the way that points outside the patient are assigned dose.

Figure 3 shows close agreement between the doses calculated using this software and those calculated using the TomoTherapy planned adaptive module. For a typical patient with prostate cancer, the mean dose difference was 0.36%, and >98% of points agreed between the two calculations with a 3%/3 mm box index,^26^ excluding points <5 mm deep in the patient (Figure 4).

**Figure 5.**
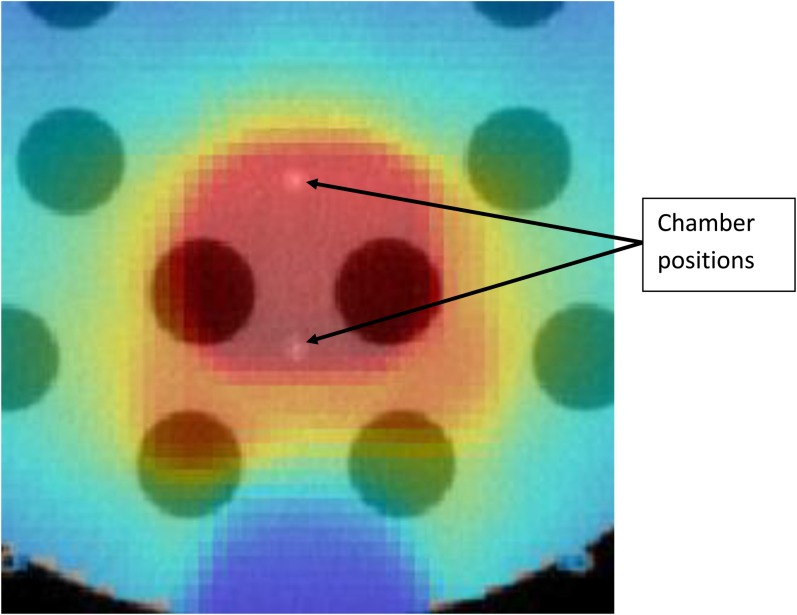
Measurement of the impact of changes in patient densities. A clinical prostate plan was recalculated on the cheese phantom, in the section of it with removable plugs. The plan was delivered three times, once with unit density plugs in all holes, once with the inner ring removed and once (as shown) with all the plugs removed. The figure shows a dose distribution superimposed on a megavoltage CT image of the phantom.

To test the ability of the software to cope with large changes in patient density, a prostate plan was delivered to the “cheese” phantom with and without the insert plugs. Ionization chamber measurements were made at the two points shown in Figure 5. The plan was delivered three times, once with unit density plugs in all the holes, once with the inner ring of plugs removed and once with all the plugs removed. Table 3 shows the agreement between calculation and measurement. Even for such an extreme case, agreement was within 3.1%.

**Table 3.** Doses measured with an A1SL chamber (Standard Imaging Inc., Middleton, WI) at the two positions shown in Figure 5, relative to the dose when all the holes are filled with unit density plugs

Chamber position	Calculated		Measured		Calculated/measured	
	Inner ring removed	All plugs removed	Inner ring removed	All plugs removed	Inner ring removed	All plugs removed
Anterior	1.075	1.141	1.058	1.116	1.016	1.022
Posterior	1.064	1.148	1.042	1.114	1.021	1.031

### Time saving

#### Daily dose calculation

For the ten patients in the study by Scaife et al,^21^ the calculation of dose was performed on an interactive system and took approximately 12 h per patient, with the number of subprojections set to the minimum of one. To have performed these calculations for 151 patients would have required over 6 months of work. For the automatic calculation of 151 patients, the calculation of dose (using 117 job slots) took 12 days.

#### Automated contouring

An experienced oncologist can contour the rectum on all slices in a scan in approximately 15 min. A 151-patient data set (of whom 23 patients have 20 fractions and 128 have 37 fractions) would take approximately 35 working weeks (assuming 7.5 h per day and 5 days per week). This is clearly not practical. The automated system takes about 2 min per scan and runs on multiple parallel job slots. With 27 job slots, the outlining for the 151 patients took 7 h.

### Equivalent uniform dose to rectum

**Figure 6.**
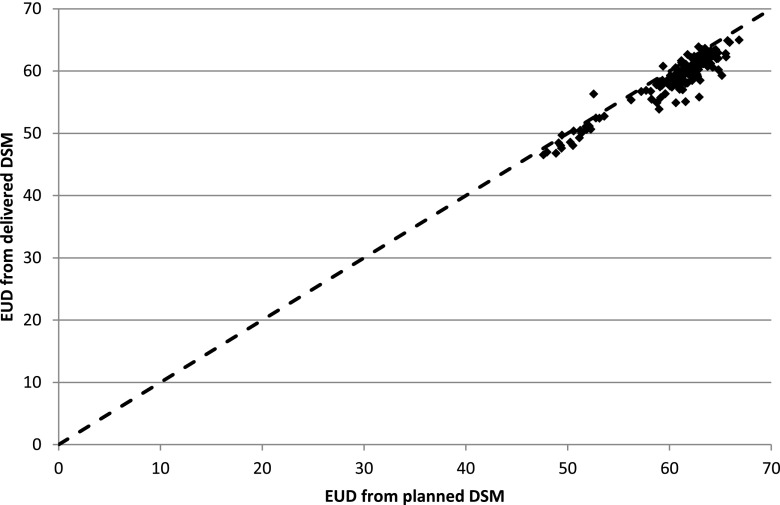
Equivalent uniform dose (EUD) to the surface of the rectum, calculated for planned and delivered dose. DSM, dose surface maps.

Figure 6 shows the results for the calculations of the EUD to the surface of the rectum. The delivered EUD is shown relative to the planned EUD for the 151 patients calculated. Although dose tracking at the voxel level remains an important goal, in practice there are many barriers in identifying structures with sufficient accuracy to achieve this. Summation of dose surface maps achieves many of the objectives of dose tracking, with fewer uncertainties. By splitting the surface at a point posterior of the centroid of each image, we ensure that the central column of the DSM corresponds to the middle of the anterior surface of the rectum. This is in contrast to the splitting at the most posterior point of each outline as described by Murray et al,^22^ which we found led to large variations from slice to slice. The anterior surface of the rectum is stable relative to the prostate and hence relative to the high dose volume in situations where daily image-guided radiotherapy is used to position the high dose volume to the prostate. Work is in progress identifying features in the delivered DSMs and linking them to toxicity.

Figure 6 shows that delivered EUD appears to be on average 3% lower than the planned EUD, in addition to large differences between patients. Further work is needed to determine whether this systematic difference results from clinical factors such as changes in rectal shape during the course of treatment or whether it results from differences between the methods of outlining on the planning CT scans and the on-treatment MV CT scans.

## CONCLUSION

We have developed a system for automatic dose recalculation of TomoTherapy dose distributions. The system does not tie up the clinically needed planning system but can be run on a cluster of independent machines. The use of the computational task-management system GANGA^10^ enables automation of the process of recalculating delivered doses, dose–volume histograms and DSMs without user intervention.

The automation, using a framework adapted from particle physics, has enabled us to scale up the dose calculation and image segmentation and to process data for hundreds of patients, rather than the tens of patients that can be achieved by manual means. Use of automatically generated input files guaranteed consistency, reproducibility and freedom from user input errors for the result data set. In conjunction with the ongoing collection of patient toxicities, this method of automatic recalculation will enable the goals of the VoxTox programme^7^ to be achieved, enabling us to compare planned dose and delivered dose with observed toxicity.

## FUNDING

JES is supported by Cancer Research UK through the Cambridge Cancer Centre. MR, AMB and KH are supported by Cancer Research UK through the VoxTox Research Programme. NGB is supported by the National Institute for Health Research Cambridge Biomedical Research Centre.

## References

[1] Chen L, Paskalev K, Xu X, Zhu J, Wang L, Price RA (2010). Rectal dose variation during the course of image-guided radiation therapy of prostate cancer. *Radiother Oncol*.

[2] Murthy V, Shukla P, Adurkar P, Master Z, Mahantshetty U, Shrivastava SK (2011). Dose variation during hypofractionated image-guided radiotherapy for prostate cancer: planned versus delivered. *J Cancer Res Ther*.

[3] Peng C, Ahunbay E, Chen G, Anderson S, Lawton C, Li XA (2011). Characterizing interfraction variations and their dosimetric effects in prostate cancer radiotherapy. *Int J Radiat Oncol Biol Phys*.

[4] Hatton JA, Greer PB, Tang C, Wright P, Capp A, Gupta S (2011). Does the planning dose–volume histogram represent treatment doses in image-guided prostate radiation therapy? assessment with cone-beam computerised tomography scans. *Radiother Oncol*.

[5] Scaife J, Harrison K, Romanchikova M, Parker A, Sutcliffe M, Bond S (2014). Random variation in rectal position during radiotherapy for prostate cancer is two to three times greater than that predicted from prostate motion. *Br J Radiol*.

[6] Jaffray DA, Lindsay PE, Brock KK, Deasy JO, Tomé WA (2010). Accurate accumulation of dose for improved understanding of radiation effects in normal tissue. *Int J Radiat Oncol*.

[7] VoxTox (2013). VoxTox—linking radiation dose at the voxel level with toxicity. An observational study to collect comprehensive toxicity data for patients undergoing image-guided intensitymodulated radiotherapy to the head and neck, prostate, and central nervous system. [Internet]. http://public.ukcrn.org.uk/search/StudyDetail.aspx?StudyID=13716.

[8] Burnet NG, Adams EJ, Fairfoul J, Tudor GSJ, Hoole ACF, Routsis DS (2010). Practical aspects of implementation of helical tomotherapy for intensity-modulated and image-guided radiotherapy. *Clin Oncol*.

[9] Langen KM, Meeks SL, Poole DO, Wagner TH, Willoughby TR, Kupelian PA (2005). The use of megavoltage CT (MVCT) images for dose recomputations. *Phys Med Biol*.

[10] Mościcki JT, Brochu F, Ebke J, Egede U, Elmsheuser J, Harrison K (2009). Ganga: a tool for computational-task management and easy access to Grid resources. *Comput Phys Commun*.

[11] Thomas SJ, Eyre KR, Tudor GSJ, Fairfoul J (2012). Dose calculation software for helical tomotherapy, utilizing patient CT data to calculate an independent three-dimensional dose cube. *Med Phys*.

[12] Yadav P, Tolakanahalli R, Rong Y, Paliwal BR (2010). The effect and stability of MVCT images on adaptive TomoTherapy. *J Appl Clin Med Phys*.

[13] Pukala J, Meeks SL, Bova FJ, Langen KM (2011). The effect of temporal HU variations on the uncertainty of dose recalculations performed on MVCT images. *Phys Med Biol*.

[14] Crop F, Bernard A, Reynaert N (2012). Improving dose calculations on tomotherapy MVCT images. *J Appl Clin Med Phys*.

[15] Langen KM, Papanikolaou N, Balog J, Crilly R, Followill D, Goddu SM (2010). QA for helical tomotherapy: report of the AAPM Task Group 148. *Med Phys*.

[16] (2011). Ganga: Gaudi/Athena and Grid Alliance. [Internet]. http://ganga.web.cern.ch/ganga/.

[17] (2013). Accuray. TomoTherapy DICOM conformance statement [Internet]. http://www.accuray.com/sites/default/files/tomotherapy_conformance_2013.pdf.

[18] Chan TF, Vese LA (2001). Active contours without edges. *IEEE Trans Image Process*.

[19] Sutcliffe M, Harrison K, Scaife J, Parker M, Romanchikova M (2015). *Auto-contouring of the rectum on MV CT images. Cambridge University Engineering Department Technical Report CUED/C-MICROMECH/TR.100 [Internet]*.

[20] Tudor GSJ, Thomas SJ (2013). Impact of the fixed gantry angle approximation on dosimetric accuracy for helical tomotherapy plans. *Med Phys*.

[21] Scaife JE, Thomas SJ, Harrison K, Romanchikova M, Sutcliffe MPF, Forman JR (2015). Accumulated dose to the rectum, measured using dose–volume histograms and dose-surface maps, is different from planned dose in all patients treated with radiotherapy for prostate cancer. *Br J Radiol*.

[22] Murray J, McQuaid D, Dunlop A, Buettner F, Nill S, Hall E (2014). SU-E-J-14: a novel approach to evaluate the dosimetric effect of rectal variation during image guided prostate radiotherapy. *Med Phys*.

[23] Buettner F, Gulliford SL, Webb S, Partridge M (2010). Using Bayesian logistic regression to evaluate a new type of dosimetric constraint for prostate radiotherapy treatment planning. *Med Phys*.

[24] Niemierko A (1999). A generalized concept of equivalent uniform dose (EUD). *Med Phys*.

[25] Thomas S (1999). Relative electron density calibration of CT scanners for radiotherapy treatment planning. *Br J Radiol*.

[26] Thomas SJ, Cowley IR (2012). A Comparison of four indices for combining distance and dose differences. *Int J Radiat Oncol Biol Phys*.

